# Lost in space - an exploration of help-seeking among young people with mental health problems: a constructivist grounded theory study

**DOI:** 10.1186/s13690-020-00471-6

**Published:** 2020-10-07

**Authors:** Katrin Häggström Westberg, Jens M. Nygren, Maria Nyholm, Ing-Marie Carlsson, Petra Svedberg

**Affiliations:** 1 School of Health and Welfare, University of Halmstad, 301 18 Halmstad, Sweden; 2 Affecta Pscyhiatric Clinic, Sperlingsgatan 5, 302 48 Halmstad, Sweden

**Keywords:** Sweden, Mental health, Young people, Help-seeking, Support services, Grounded theory

## Abstract

**Background:**

Mental health problems among young people is a worldwide public health concern. There has been an increase in mental health problems among young people in the Nordic countries in the last 25 years, particularly in Sweden. Despite this increase, international research has repeatedly shown that young people do not access or receive support when encountering mental health problems. The purpose of this study was to explore the process of help-seeking for professional support among young people with mental health problems.

**Methods:**

The study used qualitative constructivist Grounded Theory and open-ended interviews. Thirteen young people between 15 and 23, recruited from two local support clinics, were interviewed.

**Results:**

*Lost in space* emerged as the core category, capturing aspects of both the experience of self and mental health problems as well as the process of seeking and acquiring help from professional support systems. The study identified several prominent barriers for seeking and acquiring professional help for mental health problems. The young people expressed a lack of knowledge on mental health issues and support services and substantial efforts were made to try to cope with problems on one’s own. *Lost in space* involved *Drifting - trying to make sense of own experiences and struggling to cope with problems*, *Navigating* - *searching for help through multiple attempts and contacts* and *Docking - finding support with something/somebody that feels right*.

**Conclusions:**

The theoretical model sheds light on how young people with mental health problems were met with fragmented support services. Society needs to provide encompassing, youth-friendly and flexible support services, so that attempts at help-seeking are not missed.

## Background

Mental health problems among young people is a worldwide public health concern [1, 2]. International studies show that mental health problems in adulthood often originate in adolescence with half of all lifetime cases starting by 14 years of age and up to 75% of mental disorders in adulthood presenting before the age of 24 [3]. Interventions at an early stage may prevent a deterioration of symptoms and increased suffering for individuals as well as reduce further costs for society [4–6]. Previous research has repeatedly shown that young people do not access or receive support when facing mental health problems [7, 8]. There is no straightforward path from a debut of mental health problems to gaining access to support, and the process is instead characterized by complex and varied contacts as well as lengthy delays due to both attitudinal and structural barriers [5, 7–10]. Lack of support for mental health problems is troublesome since there is a strong relation between poor mental health and other negative health and developmental concerns [4, 5, 8].

Young people with emerging mental illness typically lack sufficient symptoms to meet the diagnostic criteria required for qualification for support or care despite considerable distress [5, 11, 12]. However, research on help-seeking has been geared towards young people with psychiatric illness and less towards those with milder forms of mental health problems [13]. This is contradictory, as young people with more severe forms of mental health problems are easier to identify and thus more often referred to appropriate care, whereas young people with milder forms of mental health problems often need to rely on their own resources for accessing help [13].

There has been an increase in mental health problems among young people in the last 25 years [2, 14]. Among the Nordic countries, Swedish 15-year-olds self-report the highest rate of psychosomatic health-complaints; pain, low mood, irritability, nervousness and sleeping problems, interpreted as signs of anxiety, depression and stress-related mental health problems [14]. Swedish studies of 16–18-year olds indicate that up to 1/3 of the young population report frequent symptoms such as pain, sadness, tiredness and anxiousness [15]. The increase in mental health problems among young people in Sweden is also evident in rates of suicidal attempts and suicides compared to other age groups [16]. Swedish data also show a clear increase in use of mental health services among young people between 13 and 24 [17, 18]. Despite this, young people express that they do not receive enough support or care [19]. They state that their problems are not taken sufficiently seriously and that they most often try to cope with their mental health problems themselves since support is not available and/or suited to their needs [19].

Inadequate resources, particularly for young people in need of care “in between” general psychosocial support and psychiatric treatment have been highlighted and efforts to improve young people’s access to mental health care, evident in Sweden in recent years [20]. Establishment of a new level of care named “First line” started evolving in the beginning of 2000. The purpose was to facilitate access to care for parents, children and adolescents. However, it has not been specified through which structures this should be brought available, but both regional and local authorities such as regular health care, the school and the social services have a joint responsibility in ascertaining proper facilities. By definition, primary care is considered First Line, but this is also the case for voluntary youth health centres and in some instances paediatric psychiatric specialist care [20]. Youth health centres, although common throughout Sweden, are an optional commitment at local level, lacking clear and uniform guidelines from national authorities, common rules, regulations or requirements regarding accessibility, availability and quality of care [21].

Research on the issue of help-seeking for mental health problems among young people in Swedish context is scarce, particularly research focusing on the group “in between”. Psychological distress in relation to psychiatric in – and out-patient service use has been measured over time through quantitative analyses, showing a decreasing threshold for psychological distress when seeking psychiatric care, particularly among young women [22]. Other studies have focused on aspects of young people’s service experiences of mental health care, establishing preferences of young people when utilizing mental health services [23], and factors for improving accessibility of youth health centres for mental health problems through a questionnaire distributed to users [24]. However, no study seems to have focused on the particular pathway and process of seeking help, and little focus is also on the large group “in between”, e.g. before specialist mental health services are needed. Therefore, this study focuses on the process of seeking help through the perspectives of young people, thereby deepening our understanding of their needs, enabling early intervention through support services suited to the needs of young people. The purpose of this study thus, was to explore the process of seeking professional support among young people due to mental health problems through a focus on their experiences.

## Methods

This study used the qualitative design constructivist Grounded Theory (GT) [25]. GT is an inductive method, aiming at conceptualizing patterns of human behaviour. The focus is on processes and the context in which they take place. As seeking help is a process involving both the individual and the context where the individual is situated, it was deemed appropriate to use GT as method. Also, constructivist GT permits the existence of active interaction between researcher and participants and the emphasis is on co-construction and voice over conceptualization [25]. This suited the study since most data was collected during interviews where viewpoints were shared and interpretive understanding sought. The analysis and data collection were performed simultaneously and constant reflection and memo-taking were used to enhance analytical thinking. Furthermore, this study was done in line with the view of social constructionism that theory can only offer an interpretive portrayal of the world and not an exact picture [25].

### Setting

Two local support services geared directly at young people in a city in southwest Sweden were used for the recruitment of participants; the general Youth Clinic run by the regional health care organization and the Ecclesiastic Youth Clinic run by the local Swedish church. Young people can initiate contact themselves with both services. The Youth Clinic is open for ages 13–23 and focuses on health promotion in the areas of reproductive and mental health, the latter through support and counselling. The Ecclesiastic Youth Clinic is open for young people aged 14–25 and they are welcome to seek support for any issues. Professional support through priest, deacon and behavioural advisor are available through support and counselling. Both support services are free of charge. Due to the variation of involved professionals and contexts in this study, the terms support and counselling have been used consistently through this article, encompassing both counselling, talk therapy, psychoeducation and general support.

### Participants

This study was based on data from individual interviews with young people seeking support at the general Youth Clinic and/or Ecclesiastical Youth Clinic. Staff meeting the young persons at the two support services acted as recruiters, scanning inclusion- and exclusion criteria, and presenting the young people with the first written information about the study. The inclusion criteria were; being aged 15–24 and seeking support for mental health problems. In this study, the term ‘young people’ refers to the study population at hand with individuals aged 15–24. The World Health Organization (WHO) divides young people into adolescents, the age period 10–19, and youth 15–24, with the term young people covering the age range 10–24 [26], whereas the United Nations (UN) uses the term youth and young people interchangeably for ages between 15 and 24 (United Nations) [27]. No mental health assessment was done, which meant that the perspective and experience of the young person was enough for seeking help and being included in the study.

The exclusion criteria were; currently experiencing psychotic symptoms and/or active suicidal plans. The young people were asked to fill in a form stating their interest in participating and this form was passed on to the researcher. If an interest in participating in the study was expressed, the researcher tried to contact the young person. In most instances, contact was established, and a meeting was set up where the researcher gave an overview of the study and provided time for questions. It was carefully explained that participation was voluntary and could be terminated at any time. Informed consent was obtained in two copies and preceded interviewing. A total of 13 meetings and subsequent interviews were conducted. The interviewees were 12 females and 1 male ranging from the age of 15 to 23, with 8 interviewees being 15–19 years of age, and 5 being 20–24. They were primarily students. No sampling was possible, all who expressed a persistent interest in participating were interviewed.

### Data collection

The interviews were scheduled soon after expressing willingness to participate. They were carried out at the premises of the clinic, or sometimes at a nearby psychiatric outpatient’s clinic where the first author (KHW) also worked clinically. The interviewees were asked broad, open-ended questions and allowed to tell their stories [28]. In order to explore the processes concerning young people seeking and utilizing support, themes of interest were: perceived barriers and facilitators for seeking support, self-image and identity, resources and competence as well as views on organization and the support given. The interviews lasted between 45 and 90 min, and were audiotaped and transcribed by KHW. Sampling continued until similar themes kept re-emerging and saturation was reached, and no new leads or insights emerged, the properties of the categories and the core category were thus considered dense [25].

### Data analysis

The constant comparative approach was used for analysing the data. The material was first sorted through initial coding by KHW, which entailed a close examination of the data line by line and subsequently, incident by incident [25]. Initial codes at this point were for example “wanting to be seen” and “needing somebody to talk freely to”. KHW continued with focused coding, using the most significant and frequent initial codes. Constant comparison of codes and their content properties and dimensions allowed development of theoretical codes and tentative categories, i.e. the above codes were synthesized into a theoretical code of “feeling a need for help”. Theoretical coding occurs as the researcher searches for the relationships between initial codes and describes how these relate to each other [28]. A selection of transcripts and coding was reviewed and discussed with the authors (JN, MN and PS), modifying the interview guide slightly to allow for further exploration. New interviews were compared to previous data and coding. An additional researcher (IMC) with expertise in grounded theory was involved in the analysis at this point and firstly performed an individual analysis in order to crosscheck findings, starting with line-by-line-coding, up to theoretical coding and formation of categories, which were then discussed with KHW, and subsequently the whole research team. The categories were grouped together and conceptualized to a higher theoretical level, i.e. “Feeling a need for help” was raised to “Fumbling in Life”. The three main categories and a core, encompassing category, emerged. A visual model was drafted from the main categories and the core. Memos were continuously kept and used in the constant comparative process, advancing the analysis of relationships between categories and enabling conceptualization. The authors all brought varying competencies and expertise coming from the areas nursing, health intervention and public health, thus constituting a multidisciplinary research team. The analysis was member-checked by the first interviewee at the final stage of the manuscript. The interviewee expressed recognition of the findings and had no objections to the analysis, thus no changes were done after this. The program NVivo was used to assist with data management. Participants’ names were changed in order to protect participants’ confidentiality.

## Results

### The core

**Fig. 1:**
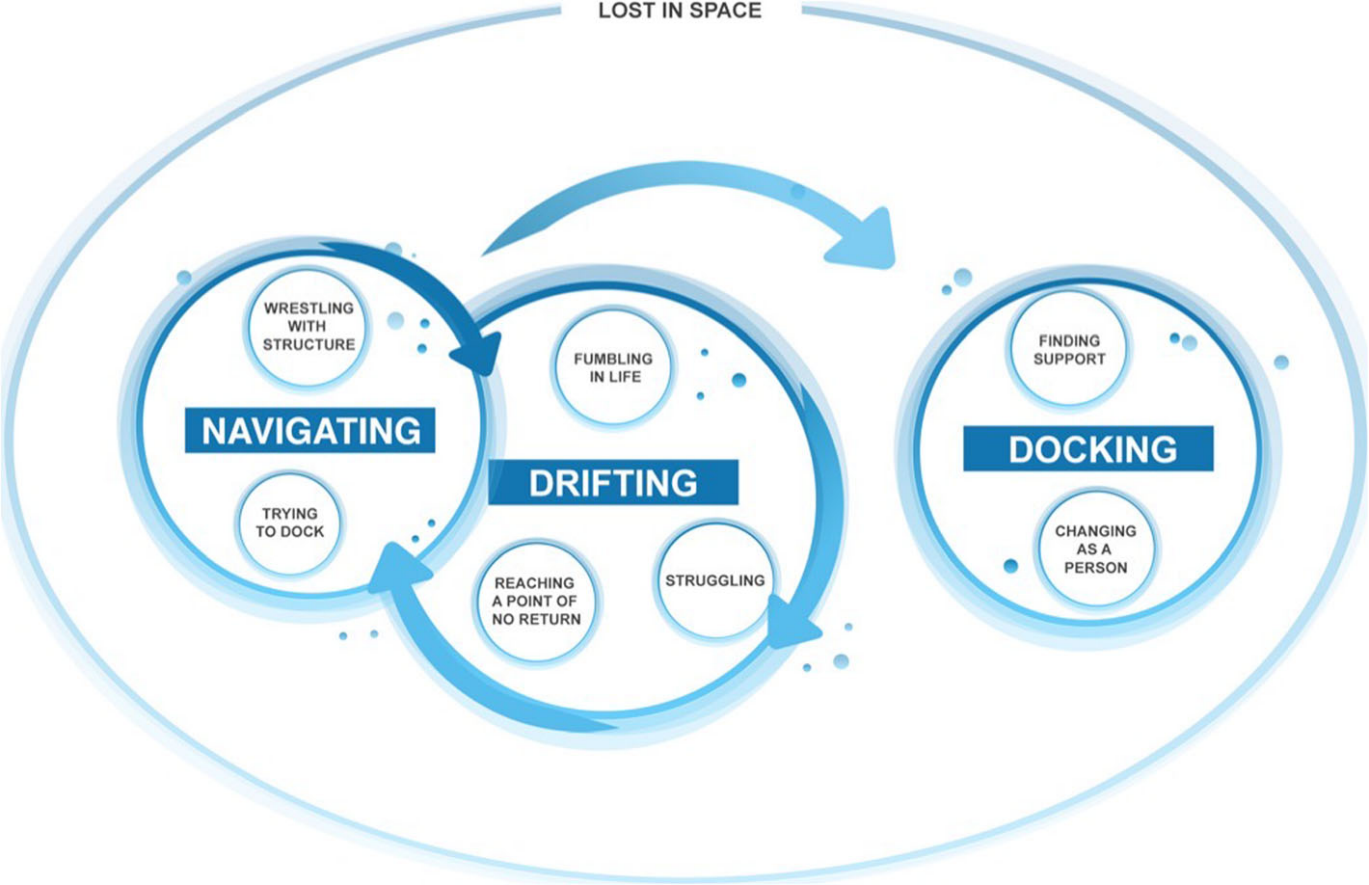
Model of the main findings of Lost in Space

Lost in space emerged as the core category, capturing aspects of both the experience of self and mental health problems as well as the process of seeking and acquiring help from professional support services. This involved three main categories; *Drifting -* the experience and unfamiliarity of mental health problems made young people drift whilst trying to make sense of own experiences. A struggle to cope with their problems was seen, using multiple strategies, eventually reaching a point of no return and seeking professional help. *Navigating* – the searching for help at various support services through multiple attempts and contacts. An inaccessibility and fragmentation of the support services made the young people navigate between different obstacles, trying to locate and access support. Encountering obstacles often meant going back to drifting before another attempt at navigating. A repetitive process of alternating between Drifting and Navigating was often seen during a prolonged period of time. *Docking –* finding support with somebody that felt right, often leading to an altered and more positive view of oneself (Fig. 1).

### Drifting

Drifting was conceptualized as an abstract category, imbued in the young people’s lives. This involved fumbling in life through unknown territory and identity forming. Drifting also contained substantial elements of struggling, trying hard to solve one’s problems through a variation of strategies, eventually, ending with a point of no return where the young person sought help for mental health problems.

### Fumbling in life

Fumbling in life encompassed aspects of both identity and knowledge of mental health problems. Thoughts on personal characteristics were compared to the feeling of having mental health problems and young people found it difficult to untangle the two, however, there was an increasing sense of not recognizing oneself. They maintained that their level of knowledge of mental health and mental health problems was too low. This was described in terms of having no idea of what was going on or where to seek help. Difficulties in assessing signs of mental health problems and not being able to describe to others what the problem was, installed a sense of insecurity. One girl expressed:

*“I suppose mental health problems are when you can’t cope with every day things … I think. But there are variations in mental health problems too. I suppose I have had and am suffering from mental health problems but I don’t know”* (female, 20).

The lack of knowledge resulted in thinking that the experienced mental health problems would not be “sufficient” for receiving support. Fumbling in life encapsulated a sense of loneliness with feelings of being different from peers. Expressions of not feeling whole and not present belonged to fumbling as well as not being able to think clearly and having part of the brain shut off. It also involved a yearning for somebody to truly see and confirm them, wanting somebody to tell them that they were ok, that they were not “too” different or sick, thus normalizing their experiences. Normalizing was done through expressing that mental health problems was a part of life, using this as rationale for the existence of one’s own problems, sometimes comparing oneself to peers or celebrities with known mental health problems. Wishing there would be a tangible, physical issue was also expressed. It was thus important to be on the right side of sanity and young people disputed being labelled (mentally) ill.

#### Struggling

Struggling explained how the young people dealt with what was perceived as a reduced level of well-being and mental health problems, a challenging and ongoing situation. Strategies were more or less continual, with a constant scanning for relief and attempts at alleviating discomfort. Young people tried strategies for changing themselves, their activities, and surroundings. It meant trying to change attitude and thoughts as well as lifestyle, schools, workplace and friends. Young people tried to find a balance between deflection and finding peace within oneself. It was considered difficult, but not impossible, to impact your own feelings and state of mind. A common strategy was shutting off by limiting social interaction, leading to isolation and a feeling of loneliness. Shutting off also manifested itself as attempts to ignore feelings or postpone dealing with them. Sometimes suppressed emotions and mental health problems would resurface and cause emotional turmoil, bouts of crying or self-harm. This in turn increased the sense of drifting, with difficulties understanding one’s own reactions. Trying different strategies resulted in a delay in seeking professional support.

Struggling encompassed a sense of ambivalence, both with regard to personal admittance of having mental health problems as well as seeking support. Talking about problems turned them into something tangible, whilst keeping the problems disclosed, made them less “real”. Making the problems visible by seeking support entailed a fear that that there was no help to be had, thus fuelling ambivalence. One participant stated:

*“I didn’t want to seek help, because I didn’t want to acknowledge that I had problems. If I say I feel bad, if I seek help and I talk about it, then it becomes something concrete”* (male, 18).

This subcategory entailed experiences of being stuck in a grey dense fog that made life unmanageable. The fog influenced their energy and ability to make decisions. All motivation was gone, there was a sadness and a feeling of lack of own worth. There was also a struggling with disappointment, not living up to own expectations on how to be as well as dealing with problems such as headaches, heartburn, anxiety and sleeping problems. A loss of function and being out of order was described in various settings such as school, work, and the household, often leading to a need for support of some kind. Struggling also contained an elevated sense of responsibility, manifesting itself as a demand to help oneself when experiencing mental health problems, finding the right help and making use of available support. Feeling better was perceived as one’s own responsibility, adding to a sense of loneliness and struggle.

*“Now I have to try and find yoga somewhere and that’s not really what I want to do right now. Feeling better seems to be my own responsibility”* (female, 22).

The young people talked of wanting to be strong, to cope on their own and conveyed a pressure of succeeding in life. Sometimes this led to them not sharing information about their own problems, specifically with parents but also close friends were excluded from the difficult experiences. This put a greater strain on the young person.

#### Reaching a point of no return

After drifting and struggling, a point of no return was eventually reached. Deterioration of symptoms, a loss of, or decreased, function would be the cue to seeking help. This was in some instances described as clear and immediate, involving a sense of being overwhelmed, frozen, overcome with anxiety, panic or sadness.

*“I felt bad the whole day. As soon as she had gone, when I came home, I collapsed and cried and had difficulties breathing and a pressure on my chest. And then I felt I’d had enough”* (female, 22).

The decision to seek professional help was often dependent on input from somebody else. This was described as somebody else taking control, often family or friends. Other “catalysts” were supportive staff in schools, midwives or church staff, coaching, guiding and supporting the young person to seek professional support.

### Navigating

Navigating was used as a depiction for the attempts of young people to seek support and what was encountered during this process. Navigating often lasted a prolonged period of time, and in some instances, years, partly due to a fluctuation of mental health problems but also due to wrestling with structural barriers, with multiple attempts of trying to dock with a functioning supporter.

#### Trying to dock

The subcategory Trying to dock described how a desire to get help led to recurring attempts at trying to gain support. Trying to dock entailed descriptions of hopes of being helped, being safe and expressions that finally, somebody would notice and help. A feeling of being engulfed in one’s own life and context called for help from the outside, where a supporter could help with guidance and an outside perspective on problems. Not knowing what was wrong, increased the feeling of needing somebody to guide you. A longing for somebody outside the family to talk freely with was expressed and a longing for being understood.

“*It felt like I would be safe, like I would get help, things would get better. Somebody will see and understand my problems and will guide me”* (female, 20).

A desire for concrete tools was not uncommon. Sometimes, seeking support for primarily physical sensations, i.e. heart palpitations or pressure on chest constituted a desire to be “seen” as not feeling mentally well. Presented problems, both emotional and physical, were sometimes not taken seriously or picked up on, leading to a sense of sadness, disappointment and worry. Many instances of unhelpful support involving communication problems were described with feelings of not being listened to, but also having problems understanding the supporter. Pointless exercises, unhelpful tips and the supporter having the wrong focus were described. The result of support not being provided as desired or in a way that felt right, was a greater determination to try one’s own strategies and/or further attempts of trying to dock elsewhere. A feeling of being unsupported or not cared for was described and the young person went back to drifting.

#### Wrestling with structure

The subcategory Wrestling with structure captured how obstacles were encountered during the process of seeking support. Waiting times, age limits or not being “sufficiently” ill were all major obstacles to seeking and obtaining support. Lack of resources and supporters recommending other support services due to waiting times was described as absurd. The notion of long queues and waiting times sometimes led to a sense of resignation. Regarding oneself as “too old”, or “too young” sometimes constituted an obstacle in approaching certain support services. Being a minor meant occasionally refraining from seeking support, being afraid of guardians finding out. Not having supportive guardians was also sometimes an obstacle for accessing primary medical care.

Various support services were described, the most common being school-staff and the general youth clinic, but other health clinics were also involved in this trial-and-error-pattern of searching for supporters. Primary care was not generally considered an option for seeking support, being regarded as dealing with physical health. Although school staff, school nurses, mentors and counsellors, were valued because of their proximity, this was double-edged since getting support in school was connected with embarrassment and an undesirable show of weakness in front of peers.

When searching for a safe docking station/support service, wrestling with structure meant having to deal with dismissals and referrals with reference to a lack of symptom gravity (not having enough problems), being on an incorrect level of care (not being sick enough or being too sick) or not the right medical speciality. This led to a sense of resignation and an impression that there was no point in trying to seek support and sent young people back to a drifting state of feeling lost.

*“I think I just felt very lost. They can’t help me here, and they won’t help me there. It was like, it was this I suspected would happen and why I didn’t bother. No one will care”* (female, 23).

Wrestling with structure meant dealing with an inadequate chain of support. Different needs were not accommodated in the same support service and this usually meant having to spread out contacts according to support type (e.g. medical advice at primary care, support and counselling at youth clinic, and physiotherapy, training, yoga etc. at private services). It also meant having to spread out contacts according to the organization of medical specialities, (i.e. one place for treating eating disorder, one for treating ADD, and another for general support and counselling.) Being forced to seek support at different services, according to a classification of having simultaneous – but different – mental health problems was described as being complicated, too much to deal with, interfering in one’s normal life and sometimes led to termination of support.

Young people expressed that they had believed that support would be more coordinated amongst different supporters and generally, tighter follow-up was asked for instead of the responsibility resting on themselves. It was sometimes described how, more or less suddenly, they found themselves without a supportive contact, due to supporter’s illness, holiday, referrals not being sent etc. This led to a negative sense of drifting and being on one’s own.

Wrestling with structure also surfaced in statements of not understanding why support structures were usually “hidden away”. Clearly signposted and visible support services were perceived as helpful, signalling an openness.

*“If the clinic had been in town, completely openly displayed, like H&M, and it says “Supportive clinic” or “This is a place for talking”, then you notice every time you pass it. That will make you remember it. That would also decrease the shame”* (female, 19).

Facilitating factors for support services were identified, such as having an easy access to support, and support being free of charge. Anonymity was greatly valued, particularly in cases where parents had not been helpful or supportive.

### Docking

Docking was the conceived space which referred to young people connecting with professional support and how the experience affected thoughts on self. This category contained information on what made contact with a supporter work in a beneficial way.

#### Finding support

The subcategory Finding support symbolized finding a seemingly safe station. This was described as ending up right but still continuing to struggle and having mental health problems. Finding support meant the feeling of loneliness decreased, simply by knowing there was somebody who cared. Young people described how they were recognized, accepted and listened to. Validation of their experiences made the drifting feeling diminish. Finding support was described as getting guidance, tools, reflections, having a place for venting and getting a perspective on what was going on. Having an outside supporter facilitated openness and constituted a place where feelings could be vented, problems disclosed and enabled feeling “pretty normal”. *“Even though you feel bad when you seek support, it feels a little bit better afterwards, because you know you will get help. You are not as lonely as you think. Somebody is there and listens to you. And that feels good”* (female, 18).

A good supporter was described as having proper education and competence combined with personal engagement. It was somebody who could help sort things out through systematic reflection. It was important that the supporter did not judge but at the same time did not become a “new friend”. There was also an issue in balancing the actual help. Young people sometimes claimed that their problems were not recognized or discarded as “usual teenage-behaviour”, sometimes however, they felt their problems were blown-up and exaggerated, leading to unwanted consequences, i.e. social services were contacted. If the support was unsatisfactory, it was often attributed to the supporter not having enough competence or training. Being a behavioural specialist, primarily psychologists, installed confidence and made the young person feel safe and supported.

### Changing as a person

Finding support contained the experience of personal change. Young people described how having mental health problems had changed them as a person. Only positive outcomes were mentioned, e.g. becoming stronger and more empathic. Stronger meant that they were not as easily affected by negativity and also that they had a possibility of helping themselves and others when encountering difficulties, expressed as:

*“Even if it was really hard, I’m not happy that it happened, but it was still good and I have learnt a lot during this period of time, about myself. So I feel that it actually turned into something positive. But I never thought something like this would happen. Never ever”* (female, 23).

Being strong also meant daring to show weakness, accepting that difficulties and stress were part of life. Changing as a person entailed gaining knowledge, both on themselves but also on mental health and support services. Young people spoke about gaining an appreciation of themselves. Being well and healthy was defined as a matter of being able to deal with issues and having both good and bad days.

## Discussion

This study interpreted help-seeking among young people for mental health problems as a process involving three interconnected categories; Drifting, Navigating and Docking. Drifting entailed aspects of both not recognizing yourself, not being able to assess mental health problems and not knowing where to seek support. Defining aberrations from your usual self is particularly troublesome for young people since they are still in an identity-forming period of life [29] and a need for venting questions and concerns of both physical and psychological character is expressed [30]. An unfamiliarity of mental health problems and support services pushed young people into self-help strategies and delayed help-seeking. The lack of knowledge thus most likely functions as a barrier to seeking support, a predicament that has been indicated in other studies, claiming that awareness and knowledge of mental health is inadequate [31, 32]. However, because help-seeking is a complex process, interventions aimed at improving help-seeking rates through educational and informational initiatives may not suffice [33].

The young people in this study struggled to deal with mental health problems and were ambivalent about acknowledging having problems. Help-seeking made mental health problems public, which in itself constituted a barrier to seek help. This is in line with other research where a tendency to deny the troublesome reality on experiences was found [34]. Normalization has been seen as part of explanation for non-help-seeking, where a circle of avoidance was used for accommodating or denying illness rather than resolving it. The young people in our study regarded help-seeking as only one of many strategies, and focus was not on avoidance but rather activity towards helping one-self. This drive could be useful when looking at accommodating the needs of young people.

The turning point for seeking professional support often coincided with another person instigating or encouraging help-seeking. This might in some instances be very helpful, however, certain groups may run a risk of not benefiting from this such as minors without supportive or resourceful guardians. The importance of socio-economic background for access to healthcare has been repeatedly confirmed, for example in how young people with immigrant background or living in neighborhoods with high levels of socio-economic deprivation being are less likely to gain access to child and adolescent psychiatric clinics through family referrals [35]. Young people who have left school or do not attend school are another group at risk of not accessing support. School has been pointed out as an important arena and gateway for providing support for young people, however since most leave school at the age of 18–19, the influence of counsellors or teachers in improving access to appropriate services for mental health problems is limited [36, 37]. Only one male young person was interviewed. This mirrors real-life, and points to how certain groups of young people have a particularly low help-seeking rate [38, 39]. This is of great concert since boys/young men simultaneously have an increased risk of low mental health when exposed to multiple socio-economic risk-factors [40]. The possible vulnerability of certain groups makes it adequate to ask not how to change help-seeking behaviour but to look at how support systems can adjust to their needs [41].

Navigating as a category entailed noteworthy findings of the fragmentation of the Swedish support services. Young people with mental health problems faced several barriers, including being required to spread out their contacts as well as devoting time and effort to locating available support services. Primary care was not generally recognized by young people as being available or an appropriate support service for mental health problems, which is in line with international literature [7]. A lack of comprehensive care was conveyed, where support had to be sought in various locations and support services. Being referred due to diagnosis, symptom-load, age or simply lack of organizational resources was common. International findings also show major structural barriers for support-seeking are; low availability of care, restricted and/or delayed access, a non-youth-friendly environment and disconnectedness of settings, each operating in a separate silo [5, 39, 42]. There has been widespread acceptance in the last decade that traditional support services are not suited to the needs of young people [12]. Large reforms are evident in some western countries with considerable structural changes of health care systems according to the principle of ‘one-stop’ multidisciplinary, integrated youth-services [8, 43]. A common denominator in these changes is the “no-wrong-door”- approach and the ambition to move from a fragmented service to a holistic, single and youth friendly service. The integrated youth services often provide both primary care, care for mental health problems, drug and alcohol services, sexual health as well as social services support [43]. A network of around 300 specific youth centres exists in Sweden. They have no state directive and no permanent funding but operate on a voluntary community or regional level. The focus of these youth centres has traditionally been on reproductive and sexual health issues. These centres are generally positively perceived by young people, however, there are big differences between groups in terms of access and widespread differences between centres in resources, staffing and competence [24]. These centres can thus not be considered an integrated youth service. Despite the fact that several Swedish reports have repeatedly established the inequality in access to support between different groups, Swedish national recommendations still focus on expanding collaboration between support services [44]. Due to mental health being a complex area that often requires efforts of a varying nature, cooperation between actors and support services is even more vital – and vulnerable and ultimately puts the young person at risk of not getting adequate support. Looking at international research and the momentum towards integrated youth centres in other parts of the world, it is worth considering whether the fragmented nature of Swedish support services for young people with mental health problems should be subject to a more comprehensive change. The ambition and willingness of young people for self-help may be encouraged but structural factors such as availability and suitability of support services are prerequisites for help-seeking.

### Implications

- Considering how young people express they have inadequate knowledge about mental health and where to seek support – initiatives for increasing knowledge on mental health and where to seek support are needed.
- Young people actively try to help themselves, a drive which may be capitalized upon – however, particular consideration for meeting the needs of groups that do not generally seek formal support is important.
- Because young people seeking support for mental health problems are met with a siloed and fragmented support system, it is worth considering a more comprehensive change of the Swedish support system.

### Methodological considerations

Quality criteria for a constructivist grounded theory may be credibility, resonance, originality and usefulness [25]. Credibility was strengthened by interviewing young people of various ages during 2 years, 2017 and 2018 at two different support services, both general health care and ecclesiastical. However, a larger sample with more interviewees and clinics, might have offered a larger variation. Several researchers were involved, performing separate coding and analysis, adding to the trustworthiness of the method. The methodological approach was thoroughly described, thus making the research process available to the reader. The study was performed in a local context with two specific clinics and may not be transferable to any setting, however, we regard the processes of help-seeking as highly transferrable to other similar contexts. Findings were in line with research from other western states, indicating the usefulness of the findings. Resonance and analytic rigour were ascertained by member-checking the analysis with one interviewee. It is however important to note, that the perspectives of young people who do not seek formal help at all, were not captured through this study.

## Conclusions

The theoretical model of this study sheds light on how young people with mental health problems were met with fragmented support services, making them Lost in space. A feeling of drifting, struggling on one’s own and navigating whilst wrestling with structure for accessing adequate support emerged. Society needs to provide encompassing, youth-friendly and flexible support services, so attempts of help-seeking are not missed. Further research is needed on how best practice models for meeting the mental health problems of young people are delivered in the Swedish context, particularly on how to meet the needs of today’s under-served groups of young people.

## Data Availability

The data generated and/or analysed during the current study are not publicly available due to participant privacy but are available from the corresponding author on reasonable request.

## Abbreviation

GT: Grounded theory
